# An Unwanted but Long-Known Company: Post-Viral Symptoms in the Context of Past Pandemics in Switzerland (and Beyond)

**DOI:** 10.3389/phrs.2024.1606966

**Published:** 2024-04-08

**Authors:** Kaspar Staub, Tala Ballouz, Milo Puhan

**Affiliations:** ^1^ Institute of Evolutionary Medicine, University of Zurich, Zurich, Switzerland; ^2^ Swiss School of Public Health, Zurich, Switzerland; ^3^ Epidemiology, Biostatistics and Prevention Institute, University of Zurich, Zurich, Switzerland

**Keywords:** historical epidemiology, long COVID, historical trends, pandemic, influenza

## Abstract

**Objectives:** Some people do not fully recover from an acute viral infection and experience persistent symptoms or incomplete recovery for months or even years. This is not unique to the SARS-CoV-2 virus and history shows that post-viral conditions like post COVID-19 condition, also referred to as Long Covid, are not new. In particular, during and after pandemics caused by respiratory viruses in which large parts of the population were infected or exposed, professional and public attention was increased, not least because of the large number of people affected.

**Methods:** Given the current relevance of the topic, this article aims to narratively review and summarize the literature on post-viral symptoms during past pandemics and to supplement and illustrate it with Swiss examples from the pandemics of 1890, 1918–1920 and later.

**Results:** Post-viral diseases were an increasingly emphasised health topic during and after past pandemics triggered by respiratory infections over the last 150 years.

**Conclusion:** In the next pandemic, it should not be surprising that post-viral conditions will again play a role, and pandemic plans should reflect this.

## Introduction

Some people do not fully recover from an acute viral infection and experience persistent symptoms or incomplete recovery for months or even years [1–3]. In the medical literature, these postviral conditions or late effects (sequelae) are collectively referred to as postviral symptoms or syndromes (PVS). The spectrum of possible symptoms is broad and often includes debilitating fatigue (postinfectious fatigue), physical weakness and exhaustion, musculo-skeletal complaints, mood swings, neurocognitive disorders (including “brain fog,” lack of concentration, etc.) or cardiovascular disturbances known to occur after acute infections with e.g., coronaviruses, influenza, Epstein-Barr, Ebola, polio or other viruses [3–5]. In analogy to post COVID-19 condition or Long Covid, post-viral disorders have the common feature that their causes are still incompletely understood and therefore therapeutic options are still limited.

A look at the past shows that PVS is not a new condition. For example, postviral fatigue syndromes have been reported in the medical literature for 100–200 years [6]. Particularly during and after pandemics caused by respiratory viruses, in which large parts of the population were infected or exposed, professional and public attention was heightened, not least because of the accumulation of affected individuals. In parallel, research on current and even past pandemics has increasingly begun to look beyond the mortality effects of pandemics to the longer-term health effects on the vast majority of survivors [7]. If historical experiences were more widely recognised, it would be apparent that the indirect effects of viruses such as influenza, polio or even SARS-CoV-2 can lead to drastic life changes, including disability, far beyond single cases [8].

Many of the post-viral symptoms mentioned above, such as incomplete or prolonged recovery or persistent symptoms such as chronic fatigue or even brain fog, have already been described in some of the survivors of previous pandemics and epidemics of the last 200 years, including the “Russian flu” of 1889/90, as well as the SARS pandemic of 2003 or the “Swine flu” of 2009. This is one of the reasons why some experts warned very early on during the COVID-19 pandemic that PVS would also play a role in this pandemic [9]. It is also known from previous long-term studies that many infections, especially those with severe courses, increase the risk of stroke, coronary heart disease or Parkinson’s disease later in life [10–15].

## Methods

There are still not many historical review articles on the history of post-viral symptoms [5, 6, 16, 17]. Given the current relevance of the topic of postviral phenomenon, this narrative and selective review article aims to contribute to this incomplete puzzle. For this purpose, we identified and narratively reviewed the international literature on the history of PVS during past pandemics. Additionally, we incorporated examples from Switzerland that we have encountered in the past 10 years of archival research on past pandemics.

## Results

### Forgotten Pandemic Experiences in Switzerland

Over the past 150 years, the world, Europe and Switzerland have been hit by various epidemics and pandemics of respiratory infections: the “Russian flu” 1889–1894, the “Spanish flu” 1918–1920, the “Asian flu” 1957–1958, the “Hong Kong flu” 1969–1970, the “Russian flu” 1977, the SARS epidemic 2003, the “Swine flu” 2009, the MERS epidemic 2012 and COVID-19 2020–2023 [18, 19]. Prior to COVID-19, the 1889, 1918, and 1957 outbreaks were the pandemics with the greatest impact on health and/or mortality, with large differences between world regions.

**FIGURE 1:**
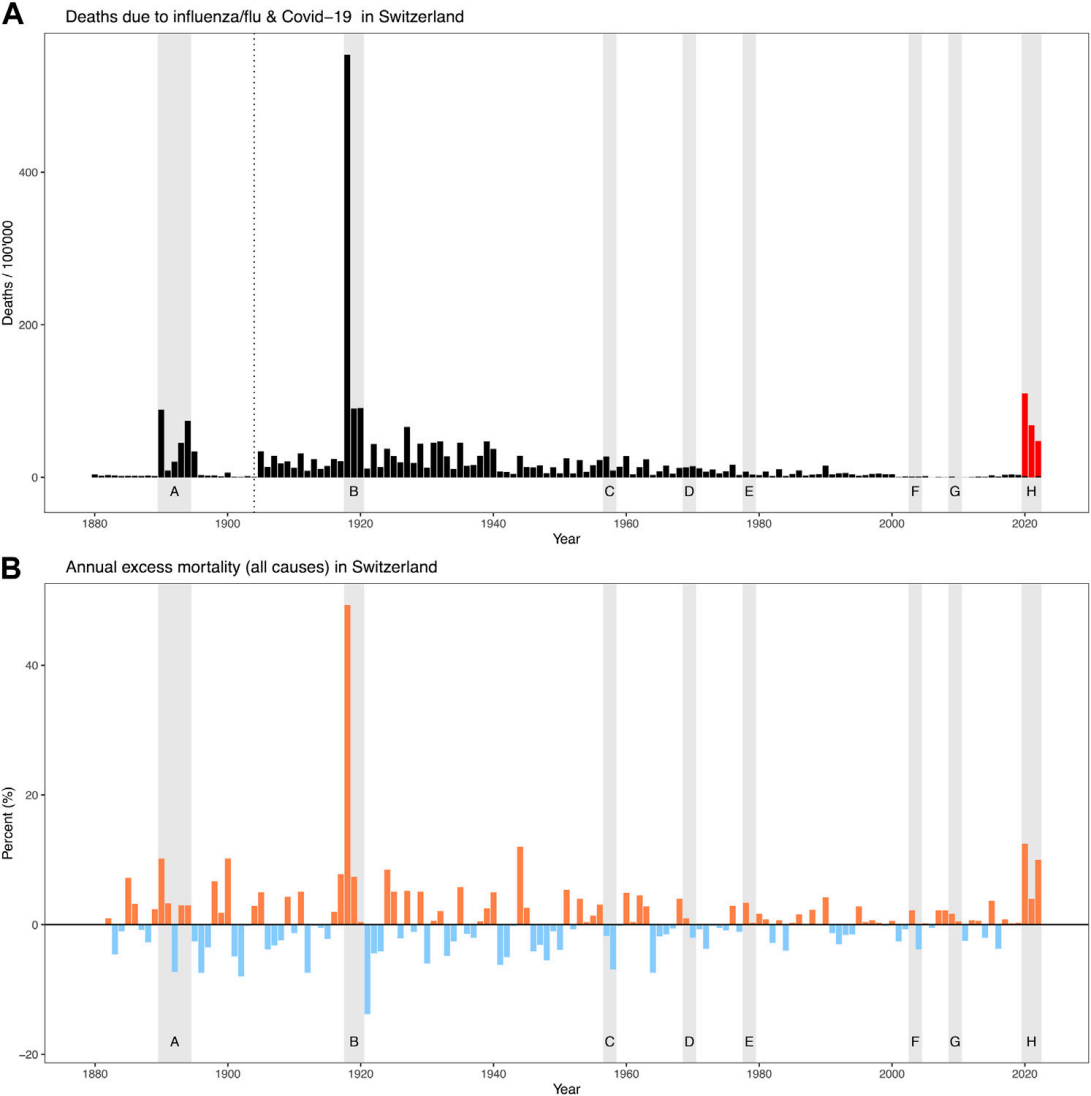
The eight pandemics/epidemic outbreaks since 1880 (gray shaded bars) and their impact on mortality: **(A)** Officially reported deaths per 100,000 population due to influenza (updated cause-of-death nomenclature as of 1904, data 1890–1894 from Schmid 1895 [28]) as well as COVID-19 (only 2020–2022 according to the Federal Statistical Office [72]); **(B)** Annual excess mortality (all causes of death) in percent (red = excess mortality, blue = lower mortality) according to [21] (figures 2021 and 2022 according to the Federal Statistical Office [73]). Pandemics/epidemics: A) “Russian flu” ca. 1889–1894, B) “Spanish flu” ca. 1918–1920, C) “Asian flu” 1957, D) “Hong Kong flu” ca. 1968–1970, E) “Russian flu” 1977, F) SARS 2003, G) “Swine flu” 2009, H) “COVID-19” 2020–2023. (Zurich, Switzerland, 2024).

In Switzerland, the “Spanish flu” of 1918–1920 remains the greatest demographic catastrophe of the 20th century [20]. It is estimated that about 25,000 people died in Switzerland from this pandemic, with the long wave in the autumn and winter of 1918 being particularly deadly. Why, as elsewhere, the 1918–1920 pandemic virus killed so many young people compared with other influenza pandemics is not yet fully understood. Comparing the various pandemics in Switzerland since the late 19th century in terms of annual (excess) mortality from all causes of death, the “Russian flu” of 1890 caused about 3000 or 10% more deaths and the “Spanish flu” of 1918 caused about 25,000 or 50% more deaths than what would be expected based on the preceeding 5 years [21]. Subsequently, however, none of the global pandemics between 1920 and 2020 reached the severity of the “Russian” or “Spanish flu” in Switzerland (Figure 1). The lack of mortality impact of recent pandemics in Switzerland and the decline in influenza-related mortality in the second half of the 20th century probably led to a loss of experiential knowledge and collective memory of pandemic disasters, which in turn led to an increasing neglect of immediate pandemic risks in the general population [22].

Where does COVID-19 rank in the history of pandemics? The recent pandemic is a historic event and led to an excess mortality in Switzerland not seen since the “Spanish flu” of 1918–1920. As in 1918, the second wave in the autumn and winter of 2020 was particularly deadly. In total, COVID-19 caused about 9,000 or 12% more deaths than expected in 2020 alone [21]. The fact that shortly afterwards, in 2022, there was again a significant increase in excess mortality of about 10% (which is at least partly caused by COVID-19) has not been observed in this way for a long time. By the end of 2022, a total of about 19,000 people had officially died from COVID-19 in Switzerland. In absolute terms, this is not far from the number of victims of the “Spanish flu” (around 25,000 deaths). However, in relative terms, as a proportion of the population, there is still a large difference (220 deaths per 100,000 inhabitants in 2020–2022 compared with 670 deaths per 100,000 inhabitants in 1918–1920). It has recently been estimated that coronavirus vaccination may have prevented about 50,000 deaths in Switzerland in 2020–2022. Thus, there is at least a theoretical possibility that, even relative to the population, COVID-19 would have had the potential to reach the level of “Spanish flu” in terms of mortality in the absence of vaccination [23].

In pandemic research, the severity of a pandemic is often measured by the number of deaths or excess mortality [19]. However, it is the nature of pandemics caused by respiratory infections that a large proportion of the population becomes ill and the vast majority survives. However, the overall burden of disease and the long-term consequences of the infections survived during previous pandemics are not well understood. There is evidence that long-term effects may not become apparent for decades. In Switzerland, for example, mortality of the 1919 birth cohort, who were *in utero* during the 1918 pandemic, showed increased mortality from the age of 50 compared with birth cohorts immediately before and after the pandemic [24, 25].

### Post-Viral Symptoms After the “Russian Flu” of 1889/90

The “Russian flu” was the first truly global pandemic in a world just connected by rail and covered by the mass media [26]. It probably originated in the Russian Empire and spread rapidly across Europe along trade routes in a matter of weeks from early December 1889. The pandemic killed around 1 million people (0.07% of the world’s population at the time) [27]. Depending on whether one assumes a single- or multi-wave pandemic, between 3,000 and 7,000 people died of influenza in Switzerland in 1889–1894, and depending on the region, up to 80% of the population fell ill [28]. The first and strongest wave swept through Switzerland between December 1889 and February 1890, but smaller to medium waves occurred repeatedly in subsequent winters until 1894. Due to the lack of human samples from this period, it has not been possible to identify the pathogen genetically. For decades, it was thought to be an influenza A virus (H2N2 or H3N8) [29, 30]. More recently, particularly in the context of COVID-19, it has been hypothesised that it may also have been a coronavirus. This theory is indirectly supported by temporal correlations of the common ancestors of today’s coronaviruses dating back to about 1890 [31] and by specific symptoms reported by physicians [32, 33]. This question will remain open until genetic evidence is available [34].

The literature contains a relatively large number of references to post-viral symptoms during and after “Russian flu,” such as persistent fatigue, cardiac problems, anxiety, neurological disorders, and so forth [35]. As early as October 2020, medical historians Mark Honigsbaum and Lakshmi Krishnan drew parallels between post-infectious neurological disorders at the time of the “Russian flu” and Long Covid based on symptom descriptions [16]. As an example of a sufferer, they cited Josephine Butler, an English campaigner for women’s rights, who fell ill with “Russian flu” during the Christmas period of 1891 and suffered from pneumonia for several days. Although her fever had subsided, she reported 3 months later that her general condition had hardly improved. In January 1892 she wrote to her son (quoted from Honigsbaum and Krishnan [16]): *“I don’t think I ever remember being so weak, not even after the malaria fever at Genoa”*[16]*.* And 3 months later, still weakened, she wrote: *“I am so weak that if I read or write for half an hour I become so tired and faint that I have to lie down”* [16].

**FIGURE 2:**
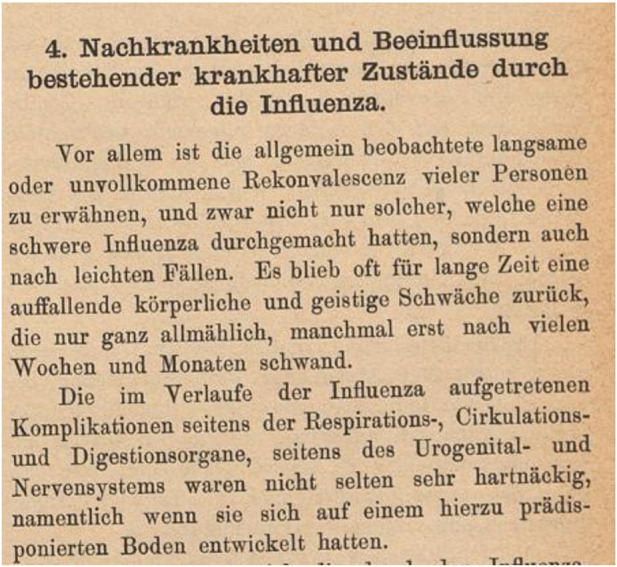
The beginning of the subchapter “secondary illnesses” in the more than 300-page report of the Swiss Health Office after the “Russian flu” of 1889–1894 [28]. (translated: *Sequelae and the influence of influenza on existing pathological conditions*—*Above all, the generally slow and incomplete recovery of many people, not only of those who had suffered from severe influenza, but also of those who had suffered from mild cases, should be mentioned. Often there remained for a long time a striking physical and mental weakness, which disappeared only very very slowly, sometimes after many weeks and months*.*)* (Bern, Switzerland, 1895).

In Switzerland, too, physicians and health authorities were concerned about the duration of the disease, its long-term implications and the sometimes long and incomplete recovery. In the aftermath of the pandemic, the Swiss Federal Office of Public Health conducted a pandemic survey among about 700 physicians in Switzerland. The result was a report of over 300 pages, written by the then director of the office, Dr Friedrich Schmid, and published in 1895 [28]. This report not only reconstructed the course of the pandemic, but also described the disease in detail on the basis of the physicians’ reports. A separate subchapter was devoted to the sequelae. It states, among other things (translated from German): *“Above all, the generally slow and incomplete recovery of many people, not only of those who had suffered from severe influenza, but also of those who had suffered from mild cases, should be mentioned. Often there remained for a long time a striking physical and mental weakness, which disappeared only very very slowly, sometimes after many weeks and months.”* (Figure 2).

The list of sequelae mentioned in the report was extensive, including nervous system disorders, neuralgia, insomnia, psychosis, hysteria, amnesia, lung disease, myocarditis, heart failure, etc. (loss of taste is not mentioned). Pre-existing conditions were often worsened by the flu. Children and schools were also affected by reduced cognitive performance after the flu. As a large proportion of the population was ill, the many absences caused by illness, e.g., in the railways and the post office, led to considerable disruptions and restrictions [36]. For example, the medical records of a spinning mill in Mollis (Glarus) showed that of 480 workers, 86 were ill with influenza for more than 3 days and 15 workers (about 3%) were absent from work for 30–55 days (almost 8 weeks or 2 months) because of the flu [28].

**FIGURE 3:**
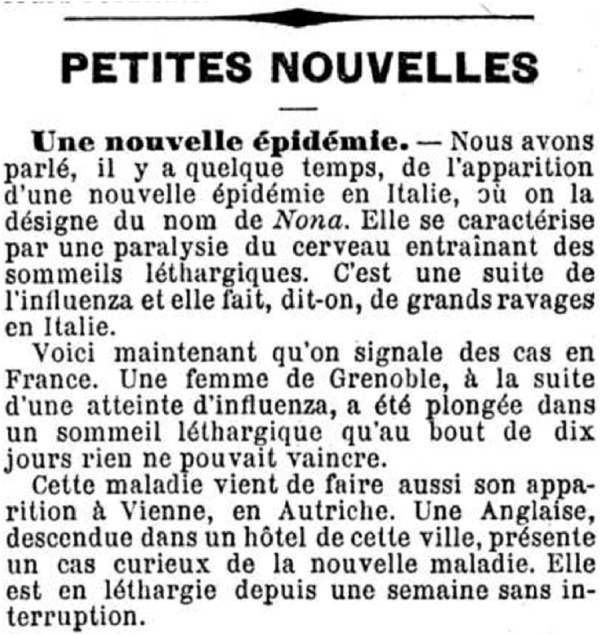
Report in the Valais newspaper “L’ami du peuple” of 12 March 1890 (page 3), on cases of sleeping sickness following the flu, a phenomenon known at the time as “Nona” *(translated: A new epidemic.*—*Some time ago, we reported on the appearance of a new epidemic in Italy, where it is known as Nona. It is characterised by paralysis of the brain, leading to lethargic sleep. It is a sequel to influenza and is said to be wreaking great damage in Italy. Cases have now been reported in France. A woman in Grenoble, following an attack of influenza, was plunged into a lethargic sleep that after 10 days nothing could overcome. This disease has also just appeared in Vienna, Austria. An Englishwoman, staying in a hotel in this city, presented a strange case of the new disease. She had been lethargic for a week without interruption). (source:* www.e-newspaperarchives.ch/?a=d&d=AMI18900312-01.2.13 *).* (Valais, Switzerland, 1890).

In the early 1890 s, several cases were also reported in Switzerland of people falling into a deep sleep, sometimes lasting for days, shortly after having contracted the flu (see the section on sleeping sickness below) [37]. As this phenomenon seemed to be spreading at the same time as an epidemic in some regions of Italy (the disease was called “Nona”) [38], Swiss newspapers feared that Switzerland might also be affected by this next epidemic (which apparently did not happen to the same extent) (Figure 3). Because of the temporal association between cases of the flu and cases of sleeping sickness, contemporaries suspected a link between the two diseases.

### Post-Viral Symptoms After the “Spanish Flu” 1918–1920

The 1918 influenza pandemic is the best-documented and researched historical pandemic and caused the most deaths, about 2.6 million in Europe alone. As the “Spanish flu” killed between 20 and 100 million people worldwide, including many young people, it is considered the “mother of all pandemics” and a worst-case scenario [39, 40]. Unlike the “Russian flu,” the pathogen causing the “Spanish flu” has been genetically identified as an influenza A/H1N1 virus [41, 42]. Although deaths were highest in this pandemic, especially among young adults, the majority of those who fell ill survived. Switzerland was hit by a total of four waves between the summer of 1918 and the spring of 1920, before the virus adopted a seasonal pattern [43, 44]. At least two-thirds of the Swiss population have fallen ill, in some cases several times, with reinfections often at least as severe as the initial illness [45].

Again, there are numerous reports in the literature of the prolonged convalescence and post-viral symptoms of survivors. One possible source is interviews with older people who survived the pandemic as children. This has been done for Ireland, among other countries, where one of the interviewees recalled (cited from Milne [46]): *“I was 5* *years of age. We all got it, all the households, there was no one moving, even the doctor who was attending us got it. … We were stricken down for 3 weeks maybe, and recovering afterwards was the most trying time of it. [A long pause.] The health services weren’t too good at the time. It was a terrible disaster.”* [46] In collections of memoirs from South Africa and New Zealand compiled by historians Howard Phillips [47] and Geoffrey Rice [48], there are also reports of post-viral symptoms such as loss of muscle strength, nervous complications, depression, apathy, tremors, restlessness or insomnia. As early as 1990 [49], Phillips described how the inability to work as a result of influenza had a considerable impact on South Africa’s economy for some time. To the north, in what is now Tanzania, influenza has even been blamed for the worst famine in a century, after a debilitating lethargy prevented flu survivors from planting when the rains came in late 1918.

It is difficult to quantify the number of people who suffered sequelae after the “Spanish flu.” According to contemporary estimates from England, out of 1,000 cases of influenza, about 800 people suffered from normal influenza with no sequelae. Of the remaining 200 people, about 70 died and about 130 did not fully recover or suffered sequelae [5, 50]. Health consequences can also occur in the medium term. In recent decades, epidemiological research has shown through long-term studies that people who survived the “Spanish flu” *in utero*, as a child or as a young adult have an increased risk of all-cause mortality later in life, as well as an increased risk of Parkinson’s disease and cardiovascular disease [15, 24, 25, 35]. There is now increasing evidence that such an association with later cardiovascular disease may also exist for COVID-19 [51].

**FIGURE 4:**
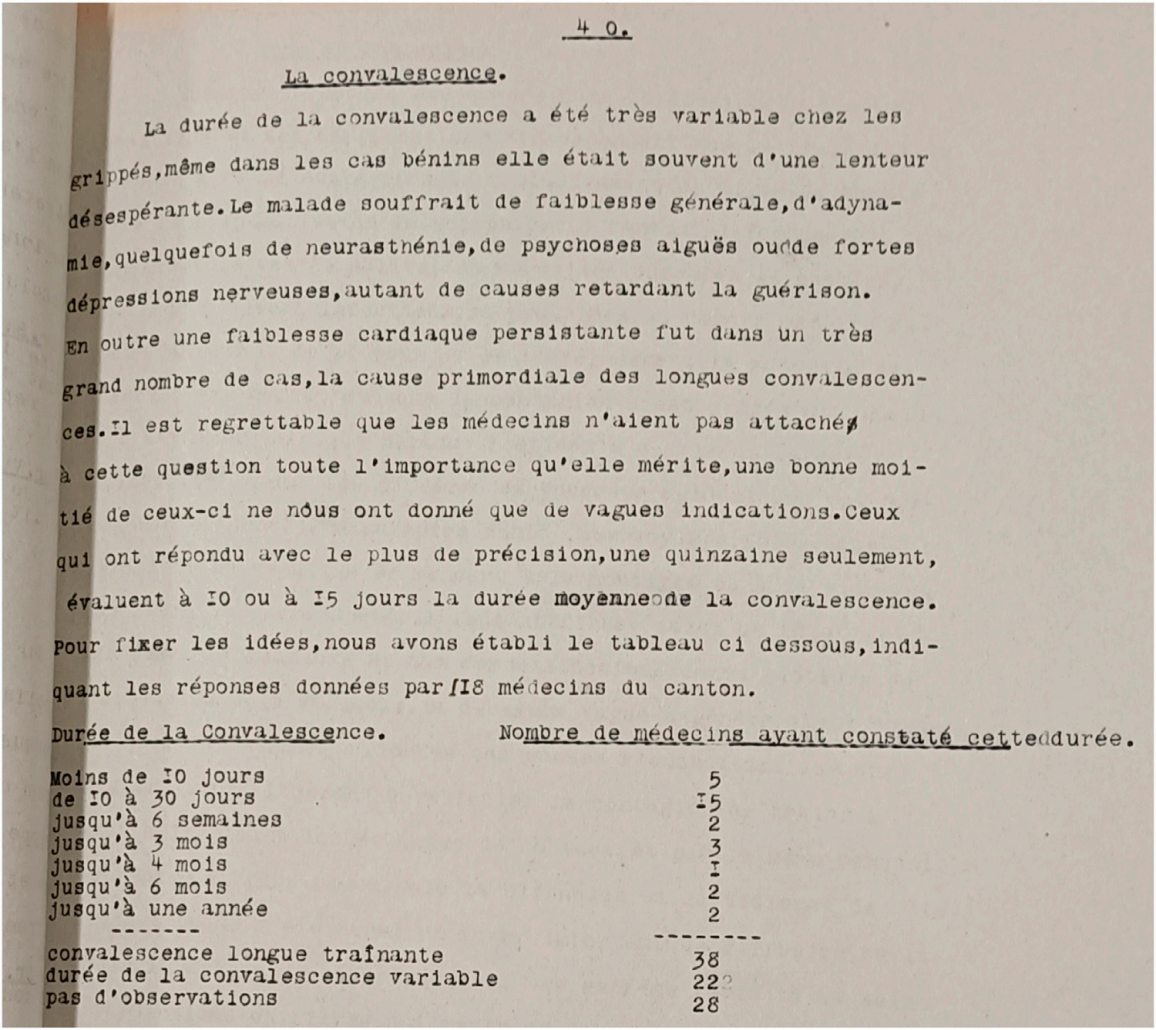
Subchapter on convalescence in the report of the Vaudois health authorities as a result of a survey of physicians after the “Spanish flu” of 1918–1919 *(translated: The duration of recovery varied considerably among influenza patients and was often desperately slow, even in mild cases. Patients suffered from general weakness, exhaustion, sometimes neurasthenia, acute psychosis or severe nervous depression, which delayed recovery. In addition, persistent heart problems were the main cause of long recovery times in a very large number of cases.) (source: Archives Cantonal Vaudoise ARC KVIIIb 27/1 & 27/2)* [52]. (Lausanne, Switzerland, 1919).

**FIGURE 5:**
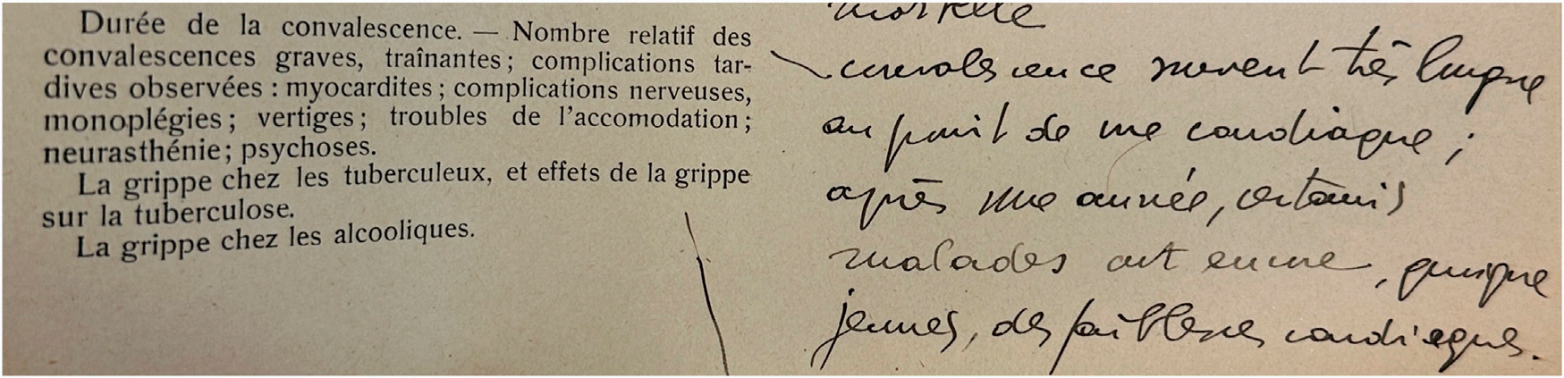
Original answer of the physician Dr. Wintsch from Lausanne to the questionnaire question about the duration of convalescence in his patient collective, the frequency of prolonged convalescence and postviral symptoms after the “Spanish flu” 1918–1920 (answer translated: “*From a cardiological point of view, convalescence often takes a very long time; after 1 year, some patients already have heart weakness at a young age”) (source: Archives Cantonal Vaudoise ARC KVIIIb 27/1 & 27/2).* (Lausanne, Switzerland, 1919).

In Switzerland, too, there are various traces of post-viral symptoms following the “Spanish flu” of 1918–1920. Once again, the health authorities attempted to reconstruct what had happened after the first waves. A survey of physicians has been preserved, this time in the canton of Vaud, where in mid-1919 the 170 physicians in the canton were surveyed with a long questionnaire about the epidemic. One of the 20 questions again concerned recovery. Questions were asked about the duration of convalescence in the treated patient group, about the frequency of prolonged and incomplete convalescence, and about symptoms and late complications. Both the individual answer sheets of the physicians and a summary report of the cantonal health authorities have been preserved in the archives [52]. The report states (translated from French: *“The duration of recovery varied considerably among influenza patients and was often desperately slow, even in mild cases. Patients suffered from general weakness, exhaustion, sometimes neurasthenia, acute psychosis or severe nervous depression, which delayed recovery. In addition, persistent heart problems were the main cause of long recovery times in a very large number of cases”* (Figure 4). Of the 118 physicians answering to the recovery questions, 8 reported convalescence periods of several months to a year for some of their patients, and another 38, without giving a time, said their patients sometimes had long and slow recoveries. The report also quotes selected responses from individual physicians. For example, Dr Décombaz of Le Sentier said that he had observed that about 15% of convalescences were long-lasting. For Dr Zbinden of Lausanne, prolonged convalescence was even more the rule. The reply of Dr Wintsch from Lausanne is shown in Figure 5 (translated from French): *“From a cardiological point of view, recovery often takes a very long time; after a year, some patients have heart insufficiency at a young age.”*

Another case study goes back to school, to the Muristalden Teacher Training College in the city of Bern, where young men were trained to be teachers [53, 54]. The Teachers’ Training College was fortunate in that it was only marginally affected by mortality from influenza in 1918 and 1919, but it was all the more affected by the burden of disease. When, at the beginning of 1919, the authorities allowed regular schooling to resume after the heavy wave of influenza in the autumn/winter of 1918, all the young men in Muristalden who had not yet contracted the disease fell ill within a few days. For four to five weeks in January and February 1919, only half the students were able to attend. About a dozen seminarians missed six to eight weeks of school because of the flu and its sequelae. The actual epidemic was over by the end of February 1919, but the health consequences were still being felt. The house chronicle states (translated from German): *“However, many of the students still needed rest and an extended holiday, and in some cases all the consequences of the flu, such as fatigue, heart palpitations, cardiac weakness and nervous phenomena, did not disappear completely until this spring, so that they had to miss some classes again.”* In addition, seminarians who had been ill with influenza for a longer period of time performed significantly worse in the intermediate exams that followed in March 1919, and the difference was still evident in the final exams 2 years later. In April 1919, the house chronicle reported (translated from German): *“In the seminary, the state of health still was not good ... For most of the term we had between two and seven inmates who were in bed. There were also heart weaknesses and all kind of relapses. One has to wonder whether these were the consequences of the flu [...].”*

### Influenza and Sleeping Sickness in the 1920 s

In addition to Parkinson’s disease (see above), the long-term neurological consequences of the “Spanish flu” of 1918–1920 also included so-called “encephalitis lethargica” or “sleeping sickness” (ICD-10 A85.8 and therefore not the same as ME/CFS) [35]. The involvement of the central nervous system can manifest as encephalitis during or after an influenza illness, whereby the symptoms usually subside without neurological consequences [55]. Post-influenza encephalitis has been described after many historical influenza epidemics, including the “Russian flu” (see above) [55].

However, in the wake of the “Spanish flu,” an encephalitis epidemic unprecedented in its frequency, virulence and consequences occurred in many countries from about 1918. The term encephalitis lethargica was first used in 1917 to describe an increasing number of patients with acute mental confusion, lethargy, fever, eye muscle movements, headache, tremors, delirium and convulsions. The lethargy that gave the disease its name lasted from days to months and in some cases led to coma and death from respiratory failure [12, 55]. In most countries, the epidemic peaked in the early 1920 s. The epidemic of encephalitis lethargica is closely associated with “Spanish flu” because of its concurrent occurrence [56]. However the exact aetiology remains unclear [12] and a causal link has not been established to this day with no influenza RNA having yet been isolated from historical brain tissue of patients [57, 58]. Because of the suspected link between encephalitis lethargica and the “Spanish flu,” it was feared at the beginning of COVID-19 that sleeping sickness might also play a role [59], but this has not yet been confirmed.

**FIGURE 6:**
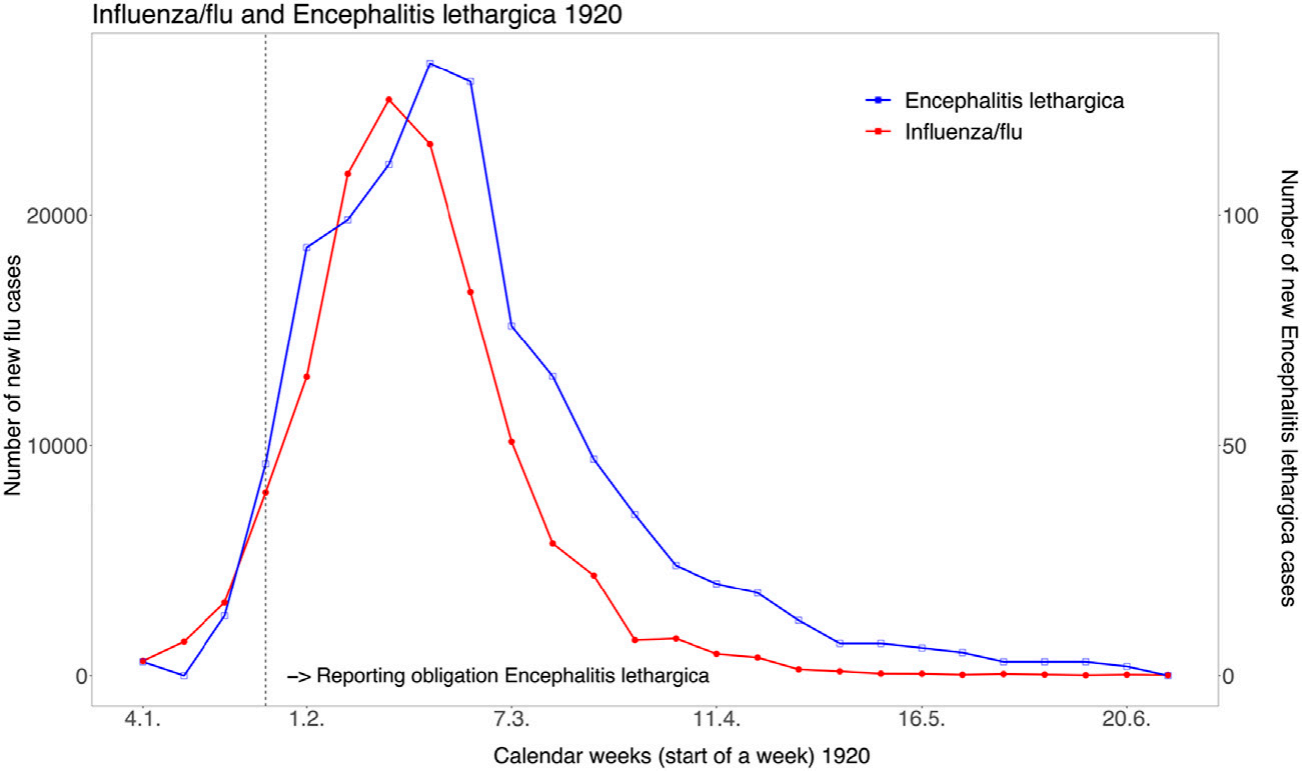
The concurrence of officially reported influenza cases and cases of encephalitis lethargica in the first half of 1920 in Switzerland *(source: Bulletin des Eidgenössischen Gesundheitsamtes, 38, 1920, p. 398).* (Zurich, Switzerland, 2024).

In the first months of 1920, many countries, including Switzerland, were affected by a so-called late wave of the “Spanish flu,” which did not reach the extent of the earlier waves, but again resulted in numerous cases of illness and deaths. At the beginning of 1920, when cases of encephalitis lethargica began to appear in Switzerland and a connection with influenza was suspected, the Federal Council ordered the compulsory reporting of this disease on 14 February 1920, and from then on the disease was systematically monitored together with influenza. In 1920 and 1921, the Federal Office of Public Health published several lengthy reports on the data generated by compulsory registration. From one of these reports in 1920, the reported cases of both diseases can be plotted by week (Figure 6). The two curves show a close temporal correspondence, with the wave of encephalitis lethargica appearing and disappearing with a delay of one to 2 weeks. In total, about 140,000 cases of influenza and 960 cases of sleeping sickness were reported between 4 January and 3 July 1920. In the 1921 report, the Federal Health Office wrote of encephalitis lethargica (translated from German): *“The coincidence of its occurrence with that of influenza—for the year 1920 the curves of the two diseases in Switzerland coincide almost completely—has led to the opinion that encephalitis is a complication of influenza. This question is still open.”* This is still true today, also because there has been no similar epidemic since.

### Post-Viral Symptoms After Other Pandemics or Epidemics Since 1920

Postviral diseases have also played a role in subsequent pandemics since 1920. This was the case, for example, after the influenza A H2N2 pandemic in the autumn of 1957, which was characterised primarily by high morbidity rather than mortality. Worldwide, the “Asian flu” in 1957 caused an estimated 1.1 million additional deaths [60]. As large parts of the Swiss population were infected or fell ill, hospitals stopped admitting visitors and schools were closed [61]. Due to the high number of cases, many companies and public institutions, such as the post office, telephone and public services, were severely affected and in some cases were only able to provide a limited service. Pensioners and auxiliary staff were deployed to make up for the lack of staff. Daily newspapers also reported major disruptions to events such as soccer championships and cycling races, where many athletes fell ill, and trade fairs such as the Olma, which attracted fewer visitors than expected. Neurological effects were also reported in Switzerland for some of those who recovered [61].

In late 2002 and throughout 2003, the SARS epidemic, the first global outbreak of severe acute respiratory syndrome (SARS) caused by the SARS-CoV virus, spread from Asia. There was only one officially confirmed case in Switzerland, but other regions of the world were much more severely affected. Again, studies show that some people have had to deal with post-viral symptoms after contracting SARS. For example, the health of recovered SARS patients was monitored after discharge from hospital, and more than half of the patients still reported severe fatigue at 3, 6, and 12 months [5, 62]. Another study followed SARS patients in Hong Kong for 4 years. 40% reported persistent chronic fatigue and 27% were diagnosed with encephalomyelitis/chronic fatigue syndrome (CFS) [63]. Based on this experience with SARS, experts warned already in 2020, relatively early in the COVID-19 pandemic, that post-viral illness would also play a role this time [9].

In 2009, the A/H1N1 influenza virus once again caused a pandemic, known as “Swine flu.” In Switzerland, an estimated 1 to 1.5 million people fell ill during the wave at the end of 2009, which lasted about 12 weeks. Not all cases were without complications; officially, there were about 600 hospitalisations and 18 deaths in Switzerland due to the pandemic virus [64, 65]. In Switzerland, there are few studies on post-viral diseases after the swine flu pandemic. In Norway, however, a study found an increased incidence of ME/CFS after influenza infection, especially in younger people, suggesting a direct link with the influenza virus [66].

## Discussion

Given this previous experience with pandemics triggered by respiratory infections over the last 150 years or so, it is not surprising that post-viral diseases are also an important issue in the context of the COVID-19 pandemic, especially after the acute phase of the pandemic, in which the vast majority of the population is likely to have fallen ill at some point. In retrospect, parallels between pandemics are not difficult to identify (and systematic archive research would probably rediscover many more past examples). However, the knowledge gained from past pandemics has been forgotten over time and was therefore too little known by health authorities and physicians at the start of the COVID-19 pandemic. Another constant over time is that although the links between the viral disease and post-viral symptoms have often been recognized, the causal mechanisms are difficult to understand and a certain degree of uncertainty remains. It should be noted that the similarities and differences in post-viral symptoms after past pandemics have not yet been fully and only unsystematically researched. It is also important to emphasise that the causative pathogens of pandemics, usually influenza viruses or coronaviruses, differ in terms of their biological characteristics, and a comparison must always take these differences into account [39].

With regard to COVID-19, it can be stated that no pandemic has ever been so well documented from a medical and biological point of view, thanks to extensive diagnostic testing, established serosurveillance programs and longitudinal cohorts set up to study and estimate the burden of Long Covid [67–69]. Pandemics will very likely continue to happen, and in the next pandemic, it should come as no surprise if post-viral diseases play a role again. In this context, the COVID-19 pandemic has underscored the importance of considering the post-acute phase also in future pandemic preparedness. This includes investing in research to further understand these conditions, planning and allocating resources to enhance post-infectious care such as the development of multidisciplinary clinics or through self-management platforms (e.g., Altea Network in Switzerland or Your Covid Recovery in the UK), implementing policies to support affected individuals to return to work or school, and developing systematic assessments and documentation to aid in social insurance and assistance when necessary (e.g., EPOCA tool of Swiss Insurance Medicine [70, 71]). Taking the lessons we learned from COVID-19 and incorporating them into our pandemic preparedness plans will be extremely crucial in reducing the long-term impacts of future pandemics on individuals and societies worldwide.
